# The Physicochemical and Antioxidant Properties of *Sambucus nigra* L. and *Sambucus nigra Haschberg* during Growth Phases: From Buds to Ripening

**DOI:** 10.3390/antiox10071093

**Published:** 2021-07-07

**Authors:** Georgiana Smaranda Marțiș (Petruț), Vlad Mureșan, Romina Maria Marc (Vlaic), Crina Carmen Mureșan, Carmen Rodica Pop, Giorgiana Buzgău, Andruța Elena Mureșan, Rodica Ana Ungur, Sevastița Muste

**Affiliations:** 1 Department of Food Engineering, Faculty of Food Science and Technology, University of Agricultural Sciences and Veterinary Medicine of Cluj-Napoca, 3–5 Mănăștur Street, 400372 Cluj-Napoca, Romania; georgiana.petrut@usamvcluj.ro (G.S.M.); romina.vlaic@usamvcluj.ro (R.M.M.); crina.muresan@usamvcluj.ro (C.C.M.); giorgiana_buzgau@yahoo.com (G.B.); andruta.muresan@usamvcluj.ro (A.E.M.); sevastita.muste@usamvcluj.ro (S.M.); 2 Department of Food Science, Faculty of Food Science and Technology, University of Agricultural Sciences and Veterinary Medicine of Cluj-Napoca, 3–5 Mănăștur Street, 400372 Cluj-Napoca, Romania; carmen-rodica.pop@usamvcluj.ro; 3 Department of Rehabilitation Iuliu-Hațieganu, Faculty of General Medicine, University of Medicine and Pharmacy, 8 Victor Babeș Street, 400012 Cluj-Napoca, Romania

**Keywords:** *Sambucus nigra* L., elderberry ripening, antioxidant activity, polyphenols, health-promoting properties, rutin

## Abstract

Elderberry growth phases represent an irreversible process involving a series of biochemical changes that have an extremely important impact on nutritional characteristics. The aim was to assess the impact of genotype and maturity stage on phenolic compounds, antioxidant capacity and mineral profile in *Sambucus* plants harvested during different growth phases, from green elder flower buds to purple-black elderberries, including pollen, peduncles and seeds. Growth phases proved to have a greater influence compared to varieties. The green buds and flowers of both varieties had a high concentration of quercetin 3-rutinoside, also termed the key compound of the study. It was found that antioxidant activity varied in the following order: blooming elder flower pollen > white elder flower buds > blooming elder flowers. Based on these findings, several novel food ingredients and supplements could be obtained in order to develop innovative health-promoting products.

## 1. Introduction

*Sambucus nigra Haschberg* (also known as standard cultivar of elderberry) and *Sambucus nigra* L. (also called European wild black elder) are some of the most versatile shrubs; native to the northern hemisphere, they belong to the *Adoxaceae* family in the order of the *Dipsacales* [1]. The comparison of major bioactive compounds and antioxidative properties of different selected types and wild plants of *Sambucus nigra* L. presents an interest in many areas, including horticulture [2], food industry [3], pharmaceutical industry [4] and, recently, in SARS-COV-2 antiviral drug research [5]. 

A new insight into elderberry anthocyanins bioactivity results from the exploring of anti-HCoV-NL63 activity of *Sambucus* Formosana Nakai stem ethanol extract and some markers of its phenolic compounds. It is has been demonstrated that *Sambucus* antiviral properties might be beneficial against the broad spectrum of human respiratory coronaviruses and applied for developing the antiviral agents in the future [6].

The chemical profile of the elderberry shrub is independently characterized whether one is talking about leaves, buds, berries, peduncles, flowers, pollen, roots, shoots, bark or seeds; although all these represent a complete life cycle of the *Sambucus* plant [7,8,9,10]. 

Fruit ripening affects several physicochemical parameters, resulting in changes in growth phases characteristics [11]. Research of the biochemical transformations underlying this process and their effects on several parts of *Sambucus* composition could provide valuable information on the stage of development of these plants suitable for formulating new food and cosmetic products.

The biochemistry of the elder fruit ripening is a genetic program that involves the degradation of chlorophyll, the biosynthesis of anthocyanins and their accumulation in vacuoles, increased cellular respiration mediated by mitochondrial enzymes and other biochemical changes [11].

Fruits of *Sambucus* commonly have a long ripening cycle. In general, one can divide the vegetation cycle in two periods, according to the most studied vegetative parts: the flower and then the fruits. *Sambuci flores* and *Sambuci fructus* present the most interest because they contain significant amounts of anthocyanins and other polyphenols [3,12,13].

The multitude of intermediate growth phases, with insufficient studies (green and red fruits, peduncles) [2] or no studies at all (elder buds), could improve the knowledge about bioactive compounds and minerals.

*Sambucus* peduncle waste resulting from the elderberry processing industry accounts for more than 10% of the total elderberry production and represents an extensive biochemical profile source of natural bioactive polyphenols with a potential low cost [1]. Actually, anthocyanins were first found in large amount in the peduncle, prior to berry ripening, and reached their highest concentrations after fruits had naturally detached. In fact, high amounts of anthocyanins compensate the xanthophyll cycle activity for decreasing the effects of light stress, throughout the *Sambucus* peduncles maturation process.

Each part of the vegetative *Sambucus* has a characteristic anthocyanin profile, mineral profile (Mg, Ca, Fe, Zn, Cu and Mn) [14] and bioactive compounds particularities, might be considered potential markers in food authenticity control. Additionally, to food implications, there is a growing interest in the use of new natural additives extracted from *Sambucus* fruits or flowers as a functional ingredient in food manufacturers, such as snacks enriched with extracts of *Sambucus* flowers, prepared by the extrusion process [15], inhibition of protein oxidation in meat and meat products with the addition of antioxidants from berries, or edible coatings [16].

Chemical composition of elder berry and flowers consists of primary metabolites (sugars, organic acids), secondary metabolites (phenols) and several other constituents (minerals, vitamins, cytokines). Secondary metabolites are susceptible to environmental and genetic growth factors and are well stated in metabolomic studies [11]. Thanks to these natural polyphenolic compounds, primarily flavonols, phenolic acids and anthocyanins, elder berry and flowers are characterized by high antioxidant activity [17]. These compounds are well known as free radical scavengers and are able to protect the human body against oxidative stress and lipid peroxidation (the literature search provided 7272 results for rutin—database PubMed).

Flavonoids were the most abundant polyphenols in elderberry fruit and were mainly represented by rutin (813.08 µg/100 g dry weight). Rutin was the most important indicator of elderberry antioxidant activity [1,12,18,19]. Rutin content in vegetative organs of plants showed a good correlation with anthocyanins content [20]. A polyphenolic bioflavonoid, the rutin (chemically a glycoside comprising of flavonol aglycone quercetin along with disaccharide rutinose) has been reported for its various rheological effects [21]. *Sambucus nigra* extract, rich in rutin, extracted from wild *Sambucus nigra* elderflowers demonstrated the protective capacity for several degenerative diseases, such as inflammatory and cardiovascular disease, cancer, diabetes and nephrotoxicity induced by gentamicin [22].

Opposed to the chemical composition of elderberry fruit, which is especially rich in anthocyanins, flowers do not contain any pigments from this group. The concentration of flavonoids, however, is the highest in elderberry flowers [23]. According to the research of Dawidowicz et al. (2006) [23] elderflowers exhibit have a much stronger neutralizing activity of free radicals compared to elderberry fruit and DPPH values between 91.95% and 94.15% were are reported. 

There is little information on the chemical composition during development among *Sambucus* species. To our knowledge, a characterization of the developmental phases from the flower bud to the ripe fruit was not made for the *Sambucus nigra* genotypes. Only few studies [2,11] were focused on pre-harvest fruit or elderberry leaves. Therefore, the aim of this study was to assess the dynamics of total phenolic content and anthocyanins accumulation, and to assess the antioxidant activity during the growth and development of flowers and fruits from wild growing elderberry (*Sambucus nigra* L.) and cultivated elderberry (*Sambucus nigra Haschberg* L.) genotypes. Additionally, the level of several minerals (Ca, Cd, Cr, Cu, Fe, K, Mg, Mn, Na, Ni, Pb, Se and Zn) was quantified during fruit development from fructification phase to ripe elderberry. In this regard, it is important to find out which of the vegetative parts of the wild/native elder could be relevant sources of secondary metabolites with high antioxidant activity possibly used in food formulations or in medicine.

## 2. Materials and Methods

### 2.1. Experimental Field and Samples

**Figure 1:**
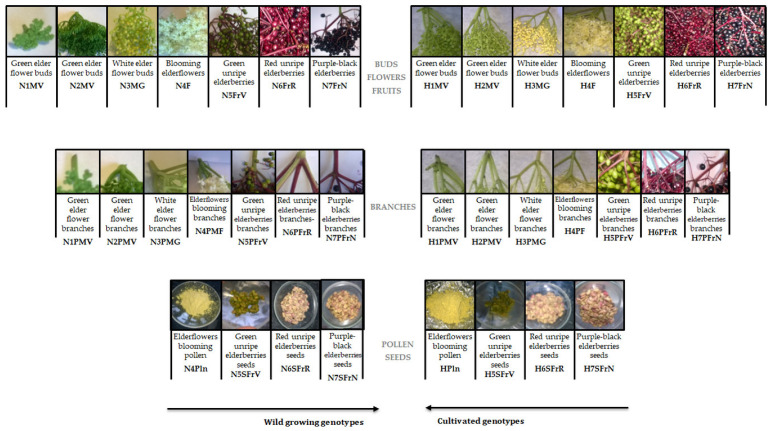
Sample coding. (H—“*Haschberg*” cultivars; N—wild “*S. nigra* L.”; M—buds; F—flowers; Fr—berries; P—peduncles; Pln—pollen; S—seeds).

Two genotypes of Romanian organic *Sambucus* samples were collected in 2016: wild *Sambucus nigra* L. from Cluj-Napoca area, and cultivated *Sambucus nigra Haschberg* L. from an orchard village of Mânău, Maramureș. Figure 1 shows the aspects of the material studied, as well as the coding of the elder samples (36 samples) at different harvesting times, starting with the green elder flower buds phase (6 May 2016) until full maturity of elderflowers blooming (29 May 2016) and ending later with purple-black elderberries (5 September 2016)—with intermediate development phases. For each ripening phase, elder samples from two species were collected between 10 and 12 a.m. Moisture content was determined according to Wu et al. [24], while ash content was determined according to Kołodziej et al. [25]. For each type and sampling phases, buds/flowers/berries clusters were harvested whole, and the peduncles were removed manually later. The seeds were separated from frozen berries (green, red unripe elderberries seeds and purple-black elderberries seeds), as previously reported by Dulf et al. [10]. All samples were stored in polyethylene bags at −18 °C until analysis. 

### 2.2. Samples Extraction for Phytochemical Analysis

The protocol for obtaining methanolic extracts from Transylvania elder samples followed the method proposed by Duymus et al. [26], with some minor modifications. The finely crushed sample of 1 g (frozen) was quickly homogenized with 100 mL of methanol and stirred using a Sigma model centrifuge model 2–5 (4000 rpm for 10 min). The extraction operation was continued by soaking in the dark, for 24 h, at a temperature of 3–6 °C. The resulting supernatant was concentrated at 35 °C under reduced pressure (Rotavap Laborata 4010 Digital, Heidolph). The extract obtained by drying was recovered in 10 mL of MeOH and stored at −18 °C. Prior to storage, the resulting extract was filtered using 0.45 μm Millipore filters.

### 2.3. Determination of Total Phenolic Content (TPC) by Folin-Ciocâlteu Method

The quantification of polyphenols was performed according to the Folin–Ciocâlteu spectrophotometric method, described by Mikulic-Petkovsek et al. [19]. For this, over 25 µL of methanolic extract, 1.8 mL of distilled water and 120 µL of Folin–Ciocâlteu reagent were added; in order to create a pH ~10, at an interval of 5 min, 340 μL of Na_2_CO_3_ solution (7.5%) was added to achieve the reaction between the phenolic compounds and the reagent used. After incubation of the samples for 90 min at room temperature, the absorbance was read using a Shimadzu UV-VIS 1700 spectrophotometer at 750 nm. The reference sample was methanol. The determination was performed in triplicate.

### 2.4. Determination and Quantification of Total Flavonoid Content (HPLC Analysis)

The concentration of total flavonoids in ripening elder samples was performed by the colorimetric method described by Diaconeasa et al. [27]. The first step was to dilute the methanolic extracts with distilled water to a volume of 5 mL and add 300 µL of NaNO_2_ (5%). After 5 min, the mixture was homogenized with 300 µL AlCl_3_ (10%) and after another 6 min with 2 mL NaOH (1 N). Then, the absorbance was read, using a UV-VIS 1700 Shimadzu spectrophotometer at 500 nm. The quantification of flavonoids content was performed using the HPLC–ESI-MS method, described by Bunea et al. [28], with some modifications. The mobile phase represented by the mixture of water and 0.1% acetic acid, meanwhile the mobile phase B consisting of acetonitrile and the same amount of acetic acid. Both of them have been subjected, on the Eclipse XDB C18 column, with gradient parameters (B from 5% to 40% (0–18 min), B from 40% to 90% (8–24 min), B from 90% to 5% (24–30 min), flow rate of 0.5 mL/minute, at 25 °C, in order to distinguish phenolic compounds from elder. The wavelengths of the chromatograms were 280, 340 and 520 nm, and the spectrum included the same values for peaks, in the range of 200–600 nm. Positive ionization with working conditions: nitrogen flow 8 L/minute, capillary voltage 3000 V, *m*/*z* 100–1000 full-scan, working temperature 300 °C. The data interpretation software was represented by Agilent ChemStation.

### 2.5. Determination of DPPH Free Radical Scavenging Capacity

The method proposed by Diaconeasa et al. [27], with some modifications, allowed the estimation of the free radical reduction capacity using a solution of 2,2-diphenyl-1-picrilhydrazyl (DPPH) and was adapted to evaluate the antioxidant activity of the different ripening elder extracts. A total of 100 µL of the methanolic extract of the samples was added to the 3.9 mL DPPH solution (0.025 g/L) to be analyzed. The mixture was properly homogenized, and kept in the dark for 30 min. The absorbance of the samples was recorded at 515 nm (UV-VIS 1700 Shimadzu). The control solution was prepared according to the same protocol but replaced the 100 μL of sample (methanolic extract) with methanol. The results were evaluated as a percentage of the inhibition capacity, based on the following equation: DPPH scavenging effect (%) = [(Ac − AP) × 100]/Ac], where: Ac—absorbance of the control solution (nm); AP—sample absorbance (nm).

### 2.6. Mineral Estimation by Atomic Absorption Spectrometry Method (AAS)

The evaluation of micro- and macro-elements (Ca, Cd, Cr, Cu, Fe, K, Mg, Mn, Na, Ni, Pb, Se and Zn) by atomic absorption spectrometry (AAS), was performed according to the method described by Pasca et al. [29]. The equipment used was the Analyst 800 spectrometer heaving a graphite furnace transverse by electric heating. The applied voltage is transverse to a tube, adjacent to the light beam, and towards the end, the electromagnet creates a magnetic field whose trajectory is parallel to the beam propagated from the lamp. The analysis sample went through three stages (drying, followed by pyrolysis and atomization), due to the rising temperature of the tube.

The mineralization of the plant matrices was performed using the calcination furnace Berghof microwave digestion system MWS-2. Amounts of 0.3 ± 0.01 g of the crushed sample were mixed with 2 mL of HNO_3_ solution (65%), followed by 15 min rest time. Before sealing the sample containers, 3 mL of H_2_O_2_ were added.

At the end of the initial program the solution was transferred at the end of the initial program in graduated containers. In the case of elder samples, the content was brought up to a gradation of 125 mL with ultrapure water. Injection of elder samples into SAA with AS-800 auto sampler was performed immediately after mineralization. Regarding the tracing of the calibration curve, calcium (2 µg/L), cadmium (2.0 µg/L), chromium (10 µg/L), copper (25 µg/L), iron (20 µg/L), potassium (5.0 µg/L), magnesium (1.0 µg/L), manganese (10 µg/L), sodium (4.0 µg/L), nickel (50 µg/L), lead (50 µg/L), selenium (100 µg/L) and zinc (2.0 µg/L) were used as dilutions of standard solutions. The results were expressed as arithmetic mean value, in mg/kg sample.

### 2.7. Statistical Analysis 

The main component analysis (PCA) was performed by The Unscrambler X 10.5.1 software. Data were reported as average mean ± standard deviation (SD) for triplicate determinations. The ANOVA analysis of variance was used to compare the average mean values, using SPSS 19.0 statistical analysis (IBM, New York, USA) and Tukey’s Honestly Significant Differences (HSD) test with a confidence interval of 95% or 99%. A *p*-value below 0.05 was considered statistically significant. The intensity of relationships between the DPPH and acid chlorogenic, flavonols values, between total phenols and flavonoids, were determined with Pearson’s correlation with a 95% confidence interval.

## 3. Results

### 3.1. Total Phenolic Content (TPC)

The differences in TPC between elderberry genotypes and the fructification stages are indicated in Table 1. The trend of the developmental stages of elderberry fruit regarding TPC varied between the samples as follows: in the early developmental phase of the bud, there was a significant value of polyphenols for both genotypes (N1MV 565.445 ± 0.003 mg GAE/100 g, H1MV 570.442 ± 0.00 mg GAE/100 g), which increased during the evolution of the bud until before the onset of blooming (883.066 ± 0.001 mg GAE/100 g N3MG, 890.04 ± 0.00 mg GAE/100 g H3MG). The elder flower blooming stage (N4F and H4F) announced a decline in TPC content; the decrease of TPC (*p* < 0.001) continued during the green unripe elderberry (N5FrV and H5FrV) and red unripe elderberry stage (N6FrR and H6FrR). 

### 3.2. Total Flavonoid Content (TFC) in Different Growth Phases of Elderberry Fruit

The profile of flavonoids in the seven developmental phases of elderberry buds, flowers and fruit (Table 1) generally indicates higher total flavonoid values in elder flower buds, while in the fruit formation stages, it involves distinct variations. Flavonoid accumulation follows the same rhythm for both studied varieties. It is important to mention the fact that flavonoids significantly contribute to a good adaptation of plants to unpredictable environmental conditions. Flavonoid biosynthetic pathways are strongly inducible, and are especially sensitive to biotic stimuli, including elicitation by bacteria [30]. White elder flower buds (N3MG and H3MG), pollen (N4Pln and H4Pln) and green elderberry peduncles (N5PFrV and H5PFrV) are of interest due to their flavonoid compound content. This study indicates the potential of bioactive compounds in the buds and peduncles of the elderberry, as unexploited plant parts.

### 3.3. Antioxidant Activity in Different Phases of Elderberry Development

The various part extracts from the wild or cultivated elderberry displayed strong antioxidant activities, especially for pollen (N4Pln and H4Pln), followed by white elder flower buds (N3MG and H3MG) and blooming elder flowers (N4F and H4F). Green elderberry seeds (N5SFrV and H5SFrV) show the weakest antioxidant action in the analyzed samples (*p* ≤ 0.001) (Table 1).

### 3.4. Phenolic Compound Profile by HPLC–ESI-MS

The concentrations of individual anthocyanins, flavonols and hydroxycinnamic acids are reported in Table 2. A total of 13 compounds were identified in the wild/native grown samples by the HPLC–ESI-MS method (similarity of the retention time and UV spectra to commercial standards), of which 11 compounds were detected in all developing phenophases: (1) cyanidin 3-sambubioside-5-glucoside, (3) cyanidin 3,5-diglucoside, (4) procyanidin dimer isomer 1, (6) chlorogenic acid, (7) catechin, (8) epicatechin, (9) procyanidin dimer isomer 2, (10) quercetin 3-rutinoside, (11) quercetin 3-glucoside and (12) kaempferol-3-rutinoside.

**Figure 2:**
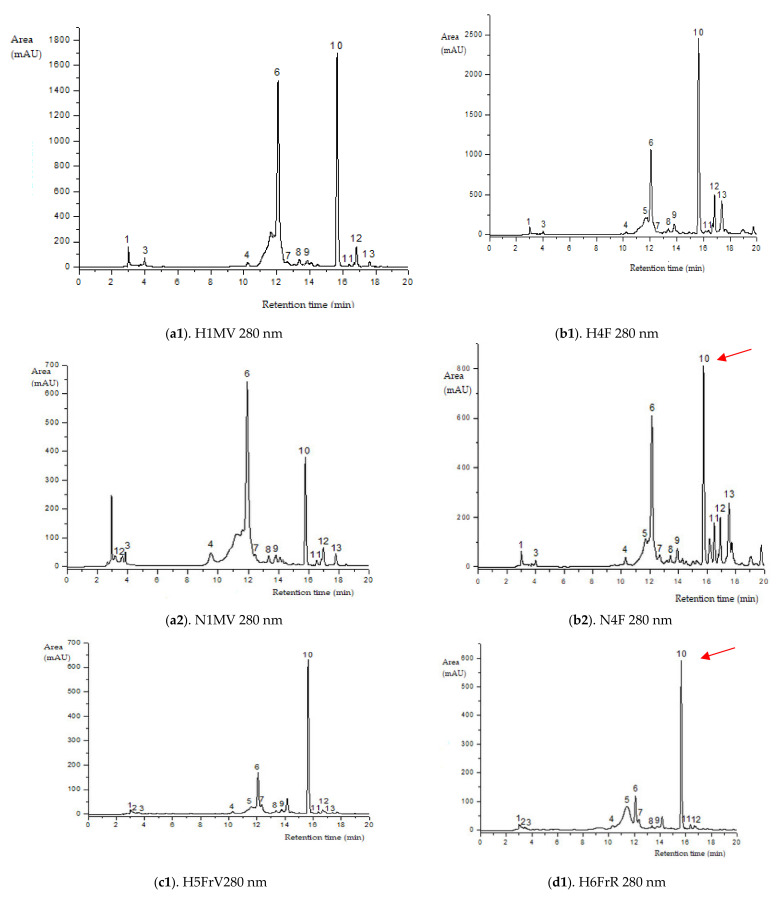

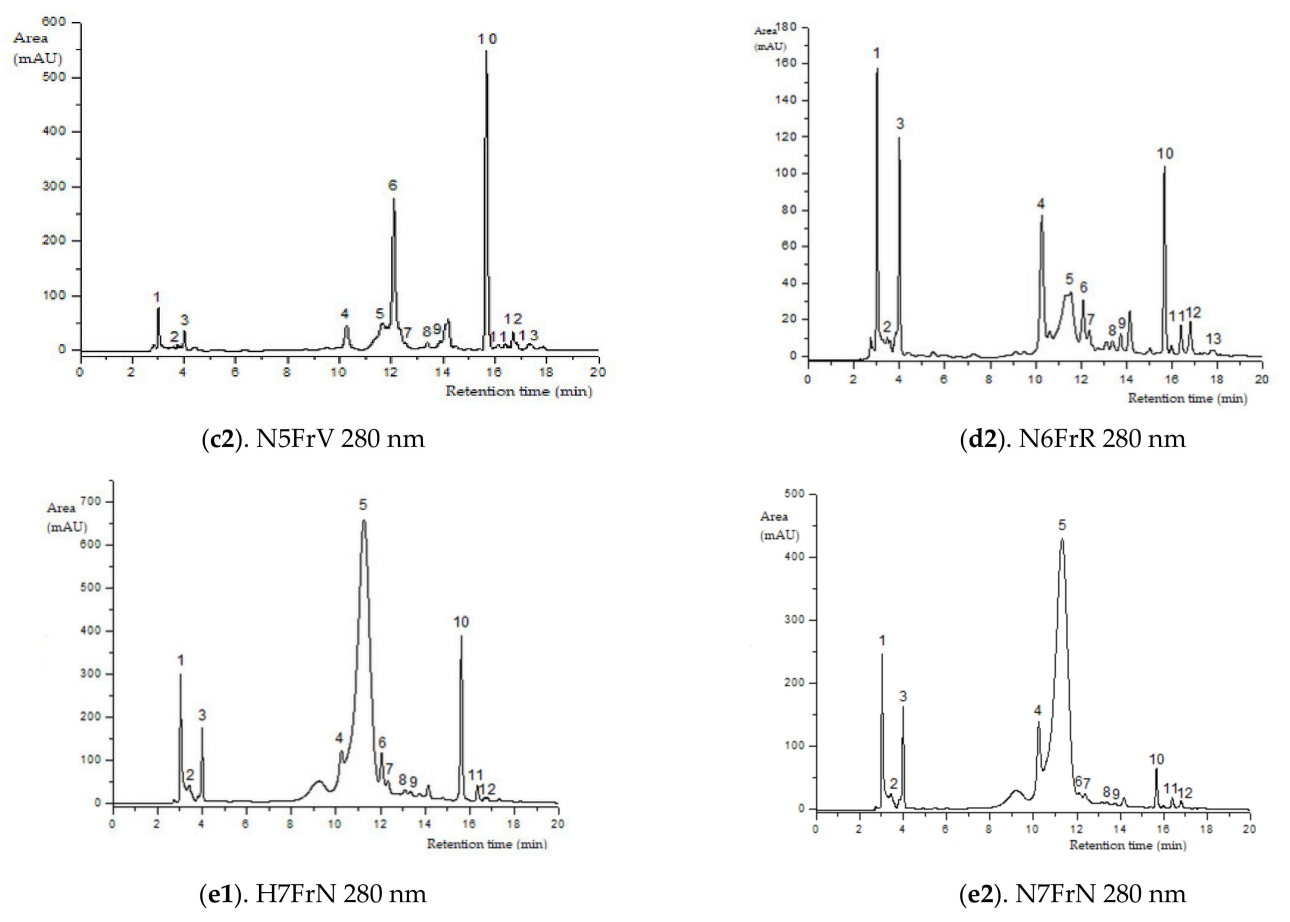
HPLC–ESI-MS chromatograms of the flavonoids content in elder samples (peak numbers correspond to those from Table 2).

The most frequent anthocyanin group in the studied elderberry varieties are cyanidin glycosides (MS2 fragmentation at *m*/*z*, 743, 581, 449, 287, Table 2, Figure 2). The representatives of anthocyanins detected in both varieties included cyanidin 3-sambubioside-5-glucoside, cyanidin 3-sambubioside, cyanidin 3,5-diglucoside and cyanidin 3-glucoside. The content of these compounds during fructification has an ascending evolution, except for cyanidin 3-sambubioside. Across the developmental stages of the elderberry, in the purple-black elderberry phase of both varieties (N7FrN si H7FrN), maximum values of anthocyanic compounds occur due to accumulation of phenylpropanoid enzymes. 

Green elder flower buds (N1MV; H1MV) (with important chlorogenic acid values), blooming elder flowers (N4F and H4F) (rich in rutin, kaempferol-3-rutinoside and procyanidin dimer isomer 3), and green unripe elderberries (N5FrV; H5FrV) (abundant in catechin, epicatechin and procyanidin dimer isomer 2) are the stages of major importance regarding the phenolic profile by HPLC of the studied elderberry varieties. Red unripe elderberries (N6FrR; H6FrR) do not show extreme values of flavonoid compounds; in contrast, purple-black elderberries (N7FrN; H7FrN) are abundant in cyanidin 3-sambubioside-5-glucoside, cyanidin 3-sambubioside, cyanidin 3,5-diglucoside, procyanidin dimer isomer 1 and cyanidin 3-glucoside.

**Figure 3:**
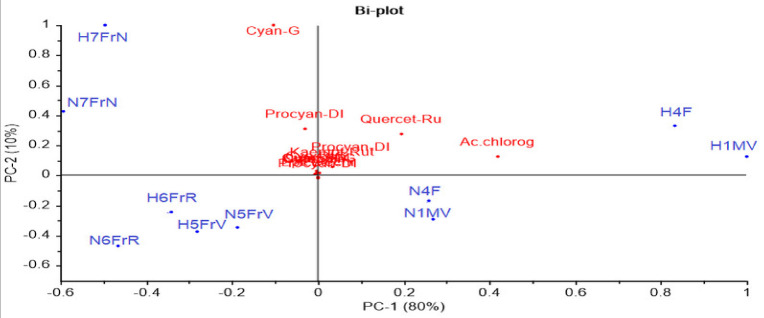
Principal component bi-plot analysis of 13 flavonoid variables measured by HPLC during ripening of elderberry. sam-5-gluc: cyanidin 3-sambubioside-5-glucoside; cy 3-sam: cyanidin 3-sambubioside; cy 3,5-digluc: cyanidin 3,5-diglucoside; procyan-DI: procyanidin dimer isomer1; cyan-G: cyanidin 3-glucoside; chlorog ac.: chlorogenic acid; catech: catechin; epicatech: epicatechin; procyan-D II: procyanidin dimer isomer 2; quercet-Ru: quercetin 3-rutinoside (rutin); q 3-gluc: quercetin 3-glucoside; k 3-rut: kaempferol-3-rutinoside; procyan-D III: procyanidin dimer isomer 3; anthocy: anthocyanins; flav: flavonols; hydroxyc ac: hydroxycinnamic acids.

To better understand the results, PCA analysis (Figure 3) was performed for the 13 flavonoid compounds identified and quantified from *Sambucus nigra* L. and *Sambucus nigra Haschberg*, in different growth phases of the fruit (Table 2).

### 3.5. Mineral Content

The analysis of the mineral content during the growth stages of the cultivated vs. wild elderberry shows extremely significant variations (*p* ≤ 0.001) in macroelements and microelements, noted in Table 3. The highest value of minerals in the cultivated variety (*Sambucus nigra Haschberg*) is in the blooming stage (H4F), followed by buds (H1MV), green elderberries (H5FrV) and red elderberries (H6FrR). In the ripe fruit stage, the wild variety (*Sambucus nigra* L.) has higher mineral element values.

### 3.6. Water and Ash

Water is an indispensable index in the quantification of technological maturity of *Sambucus* (Table 4).

## 4. Discussion

### 4.1. Total Phenolic Content and Total Flavonoid Content

TPC increases little after the ripe fruit stage has been attained, reaching for black elderberries 591.597 ± 0.00 mg GAE/100 g N7FrN and 679.254 ± 0.004 mg GAE/100 g H7FrN, which shows that the ripening process significantly influences the phenol content. Similarly to other dark colored fruits (*Rubus* L. and *Vaccinium ashei* Reade), the polyphenol content increases non-linearly with the ripening [31,32]. However, some studies [31] reported no significant differences, but these included green and red maturity of blackberry samples (*Rubus* L.) wildly grown in Turkey. Both in the wild *S. nigra* L. variety and in the Haschberg cultivar analyzed in the current study, there were significant differences in TPC due to the developmental stage of the samples and to the specificity of the two elderberry varieties analyzed.

The pollen grains in the *Sambucus* inflorescences are extremely rich in phenolic compounds (1201.126 ± 0.002 mg GAE/100 g H4Pln), flavonoid content (2100.23 ± 0.001 mg GAE/100 g H4Pln) and antioxidant activity (92.007 ± 0.00% H4Pln). 

Pollen belonging to the wild or cultivated *S. nigra* variety is an important source of health beneficial compounds, suggesting that they could be useful in preventing diseases in which free radicals are involved. Such products decrease the risk of degenerative diseases by reducing oxidative stress and metal chelating activity, in addition to their reported anticarcinogenic properties [22]. The results obtained for pollen from elder flowers are comparable to those reported for Portuguese bee pollen, where similar TPC levels are found, but with a significantly higher flavonoid content [33]. 

The values of TPC in the samples of peduncles harvested during the elder flower bud phase have ascending dynamics up to a maximum value in the green elderberry bud peduncles (442.224 ± 0.00 mg GAE/100 g N5PFrV and 429.963 ± 0.002 mg GAE/100 g for H5PFrV). During ripening of the elderberries, especially in the last maturity stages, the peduncles acquire a distinct reddish color, which is specific to the elder plant, suggesting the abundant presence of polyphenols.

During ripening, the TPC of the peduncle tends to increase from the red phase (N6PFrR and H6PFrR) to the dark color phase, and a similar tendency is seen for TPC values in the corresponding fruit. This is explained by the senescence of photosynthetic tissues in the peduncles [1]. Elderberry waste resulting from the elderberry processing industry (peduncles) could be an alternative source of unique bioactive natural polyphenols for other domains.

Additionally, in the context of using plant residues, studies indicate that seeds may contain significant levels of compounds with antioxidant and antiproliferative effects [10,34]. The total polyphenol content of red unripe elderberry seeds (57.614 ± 0.001 mg GAE/100 g N6SFrR) was similar to that of the cultivated red unripe elderberry seeds (56.241 ± 0.007 mg GAE/100 g H6SFrR). 

The results showed a striking difference between the total polyphenol content in the seeds and ripe fruit of the *Sambucus* species (Table 2). TPC in the purple-black elderberry seeds (N7SFrN) was 10.07 lower compared to the edible purple-black ripe elderberries of the wild *Sambucus* species (N7FrN). 

The literature also reports significant TPC contents in blackberry seeds (171.4 mg GAE/g extract). In that case, total polyphenols in the seed residue extracts were strongly correlated with the antioxidant activity of those fruit, suggesting that phenolic compounds contribute to increasing the antioxidant capacity value [34].

The characterization of bioactive compounds in the *Sambucus* elderberry seeds during fructification, along with the presentation of their potential beneficial properties can lead to the added value use of these seeds and fruit. More precisely, the study contributes to increasing the profitability of elderberry fruit production and processing industries, as well as of seed oil manufacturers.

Previous studies have generally compared TPC values only for *Sambucus nigra* L. flower, fruit and leave samples; to our knowledge, so far, there have been no TPC reports for buds, peduncles or pollen. *Sambucus nigra* L. flowers contain even higher amounts of phenolic compounds compared to the fruit and leaves of this species [23]. Moreover, in the case of the current study, the variety and complexity (buds, peduncles, pollen) of the analyzed developmental phases of the elderberry (18 samples/variety) demonstrate the high degree of originality, indicating increased TPC values in white elder flower buds (883.066 ± 0.001 mg GAE/100 g N3MG and 890.04 ± 0.00 H3MG), peduncles (442.224 ± 0.00 mg GAE/100 g and 429.963 ± 0.002 mg GAE/100 g H5PFrV) and pollen (1031.06 ± 0.014 mg GAE/100 g N4Pln and 1201.126 ± 0.002 mg GAE/100 g H4Pln).

The TPC results obtained for the cultivated Haschberg elderberry varieties collected from areas with a predominantly temperate continental climate suggest slight differences. Thus, TPC values in the fruit of the Austrian Haschberg variety (364 mg GAE/100 g, 510 mg GAE/100 g) [18] are lower than the values of the current study recorded for Haschberg in Romania (679.254 mg GAE/100 g H7FrN), and the wild flora samples (S. nigra) (591.597 ± 0.00 mg GAE/100 g N7FrN). In the fruit of the wild *S. nigra* L. variety in Slovenia (local population), TPC is 515 mg GAE/100 g FW [19], a result similar to that obtained for wild growing elderberry in Romania (591.597 mg GAE/100 g FW). The Mediterranean elderberry culture area seems more favorable to the accumulation of phenolic compounds in elderberry flowers and fruit [13], registering higher values compared to the temperate zone (820 mg GAE/100 g FW, 1177 mg GAE/100 g FW, 1016 mg GAE/100 g FW). Compared to the variables of the present study (different growth stages and varieties of elderberry), in other publications TPC variability in fruit is established depending on altitude [24] or the effect of the harvesting year [13].

In conclusion, significant differences of TPC between the developmental stages were observed; mainly, a decrease from the maximum TPC value of white elder flower buds (N3MG and H3MG) to the fruit formation stages (N5FrV and H5FrV), followed by a slight increase in TPC in mature fruit (N5FrN and H5FrN) was recorded. The genotype proposed for medical applications is Haschberg genotype obtained in culture, a conclusion based on the high TPC values in pollen and fruit over the last developmental stages.

Total flavonoids mainly predominate in the white elder flower bud samples (1461.262 ± 0.038 mg QE/100 g N3MG, 1369.17 ± 0.084 mg QE/100 g H3MG). Full blooming of the elder flower registers lower total flavonoid values (973.178 ± 0.001 mg QE/100 g N4F, 945.257 ± 0.001 mg QE/100 g H4F), but the pollen samples of both *Sambucus* varieties show maximum values (2131.235 ± 0.005 mg QE/100 g N4Pl, 2100.23 ± 0.001 H4Pl). Analyzing these high values of total flavonoids shows that white elder flower buds, as the complex organ of higher plants before the appearance of the flower, precede the maximum values of the flower pollen. During the full blooming phase, pollen in the elder flowers can be quantitatively diminished due to meteorological variations (wind, rain in particular), and this is also reflected in the lower value of total flavonoids.

By following the developmental stages of the elderberry, after blooming a decline in total flavonoid compounds was observed, during the formation of green elderberries (488.895 ± 0.002 mg QE/100 g N5FrV and 460.894 ± 0.002 mg QE/100 g H5FrV), a decline that continues in the red elderberry stage (304.548 ± 0.01 mg QE/100 g N6FrR and 360.53 ± 0.001 mg QE/100 g H6FrR). In fact, red elderberries have the lowest flavonoid compound value of the entire elderberry fructification process, in both analyzed varieties. Ripe elderberries (N7FrN and H7FrN) do not show considerable flavonoid compound accumulations, having only half of all flavonoids found in flowers (N4F and H4F). Compared to elderberries, elder flowers were classified as being richer in various phenolic compounds (especially flavonoids) [3,17]. The superiority of using flavonoid-rich natural extracts obtained from elder flowers over elderberries was highlighted by the qualitative and quantitative analysis (LC-ESI-MS/MS) of some corn snacks enriched with 5, 10 or 20% *Sambucus nigra* L. flower or fruit powder [15]. The results of the study above indicate that corn snacks with 20% elder flower addition represent an important source of natural antioxidants, and elder flowers can be excellent additions in obtaining functional foods.

Flavonoids can inhibit and promote iron absorption depending on their chemical structure due to chelation and/or reduction of iron [35]. However, despite the numerous studies regarding the therapeutic potential of the elderberry [4,6,22], there is no information about the possible interactions with metals, particularly the two essential trace elements, iron and copper. Due to low iron chelating activity and high iron reducing activity under acidic conditions, elderberry fruit extracts might promote iron absorption in the duodenum or proximal jejunum due to the facilitated conversion of ferric ions to ferrous ions [35]. At the same time, this study indicates that Haschberg elderberry fruit extract was the strongest metal chelator, more effective compared to standards in all tested pH conditions, but its iron reducing properties were lower than standard ones.

A statistically insignificant moderate negative correlation (r = −0.46, *p* = 0.08 ns) was achieved in our study using Pearson’s correlation test (with a 95% confidence interval) between flavonoids and iron content in samples N1MV, N4F, N5FrV, N6FrR, N7FrN, H1MV, H4F H5FrV, H6FrR and H7FrN.

Total flavonoid values in the seeds of both elderberry varieties varied similarly to those of the fruit from where they were separated. The red unripe seeds of both varieties (253.83 ± 0.012 mg QE/100 g N6SFrR, 241.329 ± 0.004 mg QE/100 g H6SFrR) had, like in the case of red elderberries (N6FrR, H6FrR), the lowest total flavonoid values. Based on the variations recorded in the analyzed samples, one may suggest that flavonoid accumulation does not depend on genotype, but only on epigenetic factors. 

The interdependence between the peduncle and the pertaining buds, flowers or fruit, regarding the presence of total flavonoids, indicates a progressive increase with the developmental stages of the elderberry, up to the maximum limit recorded in the green elderberry peduncle (986.828 ± 0.00 mg QE/100 g N5PFrV and 900.654 ± 0.00 mg QE/100 g H5PFrV). By comparing the green peduncle to the other analyzed samples, it can be seen that this might exceed even the elder flowers in terms of total flavonoid content (973.178 ± 0.001 mg QE/100 N4F, 945.257 ± 0.001 mg QE/100 g H4F). The significant values of total flavonoids, total polyphenols (442.224 ± 0.00 mg GAE/100 g N5PFrV 429.963 ± 0.002 mg GAE/100 g H5PFrV) and high antioxidant activity (39.707 ± 0.002% N5PFrV, 42.565 ± 0.004% H5PFrV) obtained in the green elderberry peduncle make these plant parts alternative sources of unique bioactive natural compounds for various industries (agrifood industry, pharmaceutical industry, etc.). 

In the fruit reddening period, red elderberry peduncles (N6PFrR and H6PFrR), ripe elderberry peduncles, respectively (N7PFrN and H7PFrN), had low total flavonoid compound values. It cannot be said that the fruit belonging to these peduncles followed the same decreasing trend of flavonoid values, because the ripe fruit (N7FrN and H7FrN) in both genotypes had increasing total flavonoid compound values towards the final maturity phase.

As expected, it was found that flavonoids were significantly positively correlated (*p* ≤ 0.05) with total polyphenols in the elder flowers, elderberries and peduncles (r = 0.679).

### 4.2. Antioxidant Activity

The most frequently associated key word with the *Sambucus* genus is antioxidant activity [1,7,12,14,17,26]. The mentioned authors indicate the antioxidant potential only in elderberry flowers and fruit (fresh, freeze-dried, pomace or powder samples), but across the developmental stages of the elderberry, according to the present study, other morphological parts with high antioxidant activity are observed (Table 1). Thus, it is important to find new sources of phytochemicals or new morphological parts of plants with high antioxidant activity (peduncles, pollen, buds) which can be used in food reformulation.

The antioxidant properties of various samples in the development of elderberry buds are attributed in the first place to the presence of anthocyanins and phenolic compounds. A directly proportional relationship was established between these compounds and DPPH antioxidant activity in the analyzed samples of both varieties. Antioxidant activity decreased from the pollen of wild and cultivated elder flowers (91.007 ± 0.000% N4Pln, 92.007 ± 0.00% H4Pln), to white elder flower buds (89.608 ± 0.001% N3MG, 90.004 ± 0.00% H3MG), to elder flowers (87.01 ± 0.002% N4F, 89.047 ± 0.007% H4F) and green elder flower buds (72.058 ± 0.033% N2MG, 70.057 ± 0.002% H2MG). Therefore, the primary developmental stages of the buds were well represented in the DPPHfree radical scavenging activity, which will increase until the appearance of elderberry inflorescences (N4F and H4F). The current study justifies the abundance of antioxidants in elder flowers, which is reported in other studies [14,23], on account of maximum DPPH values recorded in the pollen of wild and cultivated elder flowers (N4Pln and H4Pln). However, recently, a study comparing elder flowers and fruit reported an equal or higher antioxidant capacity in elderberries. This is the only study highlighting the increased potential of fruit over flowers, motivating that the growth area and the analyzed cultivar can change the rule of accumulation in different morphological parts of the elderberry [14]. Thus, antioxidant capacity is significantly influenced by the analysis technique, the growth conditions and the individual variations of the analyzed plants. It should also be emphasized that the red and green fruit of the cultivated elderberry variety (H6FrR, H7FrV) showed a higher antioxidant activity than those harvested from the wild elderberry (Table 1). Regarding the cultivated variety, ripe Haschberg elderberries had the highest antioxidant capacity (65.965 ± 0.003% H7FrN) of all the analyzed fruit samples.

Statistically significant correlations were established (*p* ≤ 0.05) between DPPH free radical scavenging activity and chlorogenic acid values in the buds, flowers and fruit of the elderberry (r = 0.711), as well as between flavonoid compounds (r = 0.725), using Pearson’s correlation test. 

The high antioxidant capacity of the elderberry is related to the total polyphenol content (Table 1); many studies confirm the relationship between polyphenol content in elderberries and DPPH free radical scavenging [7,12,14,36]. Over the past years, Portuguese elderberry peduncles have been shown to obtain antioxidant capacity scores close to those of the pertaining ripe elderberries [1]. Among the analyzed peduncle samples, the similarity of DPPH values only occurs in the case of green elderberry peduncles of the wild variety (N5PFrV) and their fruit, respectively (N5FrV). 

Elderberry seed methanolic extracts showed a reduced antioxidant capacity compared to peduncles. It can be observed, the scavenging effect of the *Sambucus* seed methanolic extract on DPPH free radicals increased with ripening: it varied from 7.723 ± 0.002% N5SFrV to 9.012 ± 0.012% N7SFrN. The same occurred in the case of seeds of the cultivated variety, 1% increases in DPPH free radical scavenging, between the developmental stages of elderberry seeds. 

Comparable to other red fruits (chokeberry, blueberry or black currant), the antioxidant activity of elderberries ranks after the maximum values of Aronia fruit (181 μmol TE/g, DPPH method; 79 μmol TE/g, ABTS method), according to the study [36].

### 4.3. HPLC–ESI-MS Characterization and Quantification of Phenolic Compositions

The anthocyanin content in ripe elderberries was higher in the cultivated variety (5235.606 ± 0.002 µg/mL H7FrN) compared to the wild variety (3545.226 ± 0.008 µg/mL N7FrN) and 17 times higher than in the green fruit phenophase (in the cultivated elderberry H5FrV). Anthocyanin abundance in ripe elderberries is also mentioned by Kiprovski et al. [2] and Mikulic-Petkovsek et al. [19]. 

Cyanidin 3-sambubioside content starts at a green bud value of 33.788 ± 0.002 µg/ml N1MV (it strictly occurs in the wild variety), disappears from the flower (both varieties N4F and H4F), reappears in the green fruit (both varieties N5FrV and H5FrV) and progressively increases with the ripening of elderberries (preponderantly higher content in the cultivated elderberry H6FrR and H7FrN). It should be noted that cyanidin 3-sambubioside and cyanidin 3-glucoside remain (they are not destroyed) in elderberry fruit-based preparations, except for fermented elderberry products [37]. Regarding cyanidin 3-sambubioside availability, this is significant even with low amounts [3]. 

The current study suggests that cyanidin 3-glucoside is the predominant pigment during the developmental stages of the elderberry. In the European Union, all colorants derived from anthocyanin are recognized as natural colorants in the E 163 classification. The interest in anthocyanic pigments has grown over the past years due to potential health benefits, including the reduction in the risk of coronary diseases, stroke, anticarcinogenic activity, anti-inflammatory effects, improved visual acuity and improved cognitive behavior [38]. 

The second important group of polyphenols includes flavonols, among which procyanidin dimer isomer 1, catechin, epicatechin, procyanidin dimer isomer 2, quercetin 3-rutinoside, quercetin 3-glucoside, kaempferol-3-rutinoside and procyanidin dimer isomer 3. The primary flavonol in the elder plant is rutin, while the other flavonols, quercetin 3-glucoside and procyanidin dimer isomer 2, occur during ripening phases in smaller amounts. 

The representative flavonols in the bud stage of the two studied varieties were kaempferol-3-rutinoside and procyanidin dimer isomer 3. Qercetin 3-rutinoside (rutin), quercetin 3-glucoside; kaempferol-3-rutinoside have higher values in the bud phase as well (N1MV and H1MV); procyanidin dimer isomer 3 increases in the blooming stage (1827.588 ± 0.002 µg/mL N4F and 2913.322 ± 0.002 µg/mL H4F). The flowers of Haschberg cultivar (H4F) contain more phenolic compounds compared to the wild variety (N4F) and also compared to its fruit (H7FrN).

*Sambucus nigra Haschberg* also continues to rank first regarding flavonoid compound accumulation in the green elderberry stage (H5FrV). Catechin (396.994 ± 0.002 µg/mL), epicatechin (205.707 ± 0.002 µg/mL) and procyanidin dimer isomer 2 (239.426 ± 0.002 µg/mL) are predominant in green Haschberg elderberries (H5FrV).

Procyanidin dimer isomer 2, rutin, quercetin 3-glucoside, kaempferol-3-rutinoside, catechin contents become particular to the elderberry blooming phase, where an increase of each mentioned compound is followed by a decline during the subsequent fructification phase. The exception is catechin variation in the cultivated variety. The content of procyanidin dimer isomer 3 was quantified 2913.322 ± 0.002 µg/mL in the flowers of Haschberg genotype. Procyanidins were the main flavonoids in the early developmental stages of the elderberry in both genotypes. During the elderberry fruit ripening stage, procyanidins were present only in the red unripe elderberry samples from the wild area (N6FrR). It was found that procyanidin dimer isomer 3 in the Haschberg variety decreased 27 times with ripening, reaching in green elderberries the value of 106.498 ± 0.002 µg/mL H5FrV, compared to inflorescences (H4F). Towards the end of the analyzed stages, procyanidin dimer isomer 3 in the cultivated variety became undetectable in the red (H6FrR) and purple-black elderberry phase (H7FrN) (Table 2). Additionally, in the wild S. nigra variety, the amount of procyanidins decreased to an undetectable level before anthocyanin synthesis. During the elderberry fruit maturity stage, the flavonoid content changes and each compound has its individual presence/disappearance pattern.

The phenolic profile during elder ripening by HPLC indicates the fact that red elderberries produce higher amounts of cinnamic acids and flavonol glycosides in fruit, particularly in the Haschberg variety. The cultivated variety is also higher in total phenolics compared to the wild genotype. In investigating hydroxycinnamic acids in the two genotypes, a linear correlation was found between total polyphenols, total flavonols, total hydroxycinnamic acids and antioxidant activity [36].

The main phenolic compound group during elder ripening was represented by hydroxycinnamic acids, the most important of which being chlorogenic acid. Other hydroxycinnamic acids found in *Sambucus* include, among others, neochlorogenic acid, cryptochlorogenic acid, 3- and 5-feruloylquinic acid and dicaffeoylquinic acids, including notably 1,5-di-caffeoylquinic acid [3] or caffeic, ferulic, p-coumaric acid [36]. The bioavailability profiles of chlorogenic acids suggest that they are metabolized mainly by the colonic microflora [39]; phenolic acids can be present as such in the majority of foods.

Chlorogenic acid was also detected in high amounts until close to the red fruit phase, when it showed a decline (884.757 ± 0.002 µg/mL H6FrR). Similarly to the analysis of unripe elderberry fruit of Ljubostinja species, regarding chlorogenic acid, Kiprovski et al. also detect significant values [2]. The preparations of different elderberry products at 100 °C for a moderate length of time (less than 30 min) showed an extreme chlorogenic acid decrease [37]. 

The two main components explained 90% of the total variance; however, no clear discrimination between the wild and the cultivated genotype was identified. As can be noticed in Figure 3, the developmental phases proved a higher influence as compared to cultivars; for example, the green buds (H1MV and N1MV) and flowers (H4F and N4F) from both cultivars registered high concentrations of quercetin 3-rutinoside and chlorogenic acid. In addition, purple-black fruit samples (H7FrN and N7FrN) showed the highest cyanidin 3-glucoside, procyanidin dimer isomer 1, cyanidin 3,5-diglucoside, cyanidin 3-sambubioside and cyanidin 3-sambubioside-5-glucoside concentrations. 

Some differences in the flavonol and phenolic acid content can be explained by the variety, being closely related to the growth conditions, the degree of maturity, handling after storage or sample preparation treatments.

### 4.4. Rutin as a Key Sambucus TPC during Growth Phases

Quercetin 3-rutinoside (rutin) was the representative flavonol in the developmental stages of the studied elderberry varieties. In the Haschberg variety, the highest rutin value (6178.463 ± 0.002 µg/mL H4F), corresponding to the elder flower blooming stage, vas recorded. This rutin value confirms the contribution of the selection of the *Sambucus* genus to the detriment of the wild elderberry (>53% of total flavonol glycosides). Regardless of the measurement method, high rutin concentrations (approximately 50 mg/g per air-dried material) are found in Haschberg flores Sambuci in other studies as well [40]. The elder flower blooming stage becomes important because elder flower extracts contain important antioxidants, with potential benefits for human health [22]. However, the variety certainly influenced total rutin content, because both the flowers (H4F) and the buds of the cultivated elderberry (H1MV) had a higher rutin content than the wild variety.

During the development of the elder plant, the variation in rutin, for each elderberry variety, is due to its synthesis, more precisely enzymatic changes. Table 2 confirms that its synthesis starts in the green elder flower bud phase (N1MV and H1MV), suddenly increases in flowers (N4F and H4F), registering a decline after the green elderberry phase (N5FrV and H5FrV). Rutin values were higher in all developmental stages of the fruit in the cultivated variety compared to the wild variety. 

Green elderberries (H5FrV) contain twice as much rutin compared to ripe fruit (H7FrN), in the case of the cultivated variety. Rutin is not the predominant phenolic compound in ripe fruit (N7FrN; H7FrN). Authors such as Thomas et al. and Dominiquez et al. [12,41] describe ripe elderberries as being rich in rutin, without relating their value to the content of other developmental stages of the vegetation cycle of the elderberry shrub. More precisely, the rutin values mentioned in other publications for ripe fruit belonging to the Haschberg variety were 72.7 mg rutin 100 g^−1^ [18] or 52.02 ± 2.48 mg CGE/100 g FW [42]. 

A parallel to the data obtained in the case of rabbiteye blueberry varieties cultivated in Guizhou (China) regarding phenolic compound accumulation during ripening shows that rutin tends to increase [32]. In contrast, rutin content decreases with the reddening of the fruit (N6FrR; H6FrR). According to the mentioned publications that study other plant organs of the elderberry shrub, such as the woody stems, green stems and leaves of *Sambucus* [2,43], rutin was also identified among the antioxidant compounds of the samples concerned. There is a currently increased interest in new plant extracts or unexploited plant parts, with a high flavonoid content. It was found that rutin is a source of biologically active phytochemicals, which can be used for the treatment of various disorders such as osteoporosis, hemorrhagic diseases, cardiovascular diseases, inflammation and cancer [4,5]. The previously mentioned studies also report that rutin has a significant pharmacological profile and could be used as a drug for the treatment of different types of dysfunctions.

In order to identify new natural sources of rutin, the green fruit of the cultivated elderberry (1504.402 ± 0.002 µg/mL, H5FrV) can be an example, due to the high rutin values during the fructification stage (green, red and black). The stage of immature elderberries corresponding to the green color is followed by a marked decrease in rutin towards the stage of mature black ripe fruit (H7FrN). The concentration of this flavonol was lower in red or black elderberries than in unripe green fruit, probably due to the rapid conversion to anthocyanin, a compound that is accumulated during these stages. Similarly to the physiological mechanisms of flavonols found in *Rubus* sp. *cousin. Loch Ness.* in three maturation stages (green, red, black), flavonols decrease in the subsequent ripening stages [30]. The formation of anthocyanin is limited in many specific plant tissues and occurs during the explicit growth phase. The clear accumulation of these bioactive compounds usually provides clues about the biosynthetic catalysts running in the pathway. Following the phenylpropanoid pathway, anthocyanins can be synthesized [30]. Biosynthesis of anthocyanins requires both genes that directly participate in their formation and genes that control the transcription of the first genes.

Starting from the dispute in the biochemistry literature that an aglycon and its associated glycoside would act similarly [44], rutin as a major compound in the flowers, buds or green fruit might act pharmacodynamically with health promoting properties. 

A rutin-rich *S. nigra* L. extract (463.20 ± 0.00 µg/mL), administered daily in 1 mL volume in vivo, proved to replace the activity level for superoxide dismutase (SOD) and catalase (CAT) at a value comparable to that of the control group; most of the anthocyanins in the plant extract were produced before penetration into the blood [22]. Rutin was also used as an ingredient for the treatment of diabetes and obesity, because it blocks the capillary walls, increases capillary flexibility and anticipates red blood cell and plasma infiltration outside the vessels, all of which due to its antioxidant property [17]. Rutin has an excellent potential even through its conversion to isoquercitin. Recently, its connection to the most widely used biodegradable and renewable thermoplastic polyester (PLA) was tested [45]. PLA is one of the biopolymers that has gained high interest with the aim of replacing polymers from inexhaustible sources. That study showed the fact that stabilizing polyesters by adding plant flavonoids significantly increased the oxidation resistance of both polylactide and polyhydroxyalkaonate.

Rutin was the major compound during the industrial processing of elderberry fruit as well. Rutin identified in S. nigra Vojsko products (Slovenia) (liqueur, juice, tea or elderberry jam) presented values from 2 to 65 mg/kg, according to Senica et al. [37]. In the same line of thought, Mlynarczyk et al. [3] reported that in elderberry pomace, phenolic compounds, rutin in particular, represent the major component (6–14 mg/g DW). 

Furthermore, a recent study focusing on leaves and unripe fruit of the wild flora (from Fruṧka Gora mountain, Serbia) and cultivated elderberry genotypes reveals the fact that the most important polyphenolic compounds are anthocyanins, with the predominance of quercetin 3-rutinoside (1–3% DW in leaves and 0.5–0.8% DW in fruit) [2]. 

Together, these approaches demonstrate that rutin in the structure of various parts of the *Sambucus* shrub is a key natural compound, a viable alternative to synthetic rutin. Flavonoids found in plant tissues represent in fact a secondary antioxidant defense system due to different stresses. Lipid peroxidation can be the result of oxidative stress that affects the integrity of the plant cell membrane. In this sense, rutin interacts with the polar head of phospholipids at the lipid water interface, increasing membrane rigidity and, consequently, protects the membranes against oxidative deterioration [44]. The relation of the rutin structure function is the representation of major biological activities.

Consequently, the flowers (H4F) and green buds (H1MV) of the Haschberg variety had the best bioactive properties of the studied samples, particularly due to the presence of rutin. Therefore, new identification of plant sources of rutin can take the form of promising dietary supplements. 

### 4.5. Mineral Content

There are no available data in the literature regarding the profile of minerals during the development of the elderberry; however, the elderberry fruit, flowers, leaves or stems have been distinctly characterized [14,46]. The biological material in the samples of the early developmental stages of the elderberry (N1MV, H1MV, N4F and H4F) is the richest in macronutrients (Ca, K, Mg and Na), particularly in green fruit, having a high Na content (N5FrV). Thus, the buds of the cultivated elderberry are extremely rich in Ca (707.229 ± 0.001 mg/kg H1MV), the flowers of the wild elderberry are rich in K (438.245 ± 0.000 mg/kg N4F) and Mg (27.966 ± 0.000 mg/kg H4F), and green fruit are rich in Na (209.725 ± 0.000 mg/kg N5FrV).

There is an extremely significant variation of calcium (*p* ≤ 0.001) in the Haschberg variety, which reaches the maximum value in the green elder flower bud phase (H1MV), also having high values in flowers (H4F) and green fruit (H5FrV). For the same elderberry variety, Młynarczyk et al. [14] focused on the idea that flowers are richer in calcium (2724.7 µg/g dry weight) compared to the fruit of the plant. Therefore, in the flower development phase, calcium was present in high values, according to the mentioned study, but the current research presents significant values starting with elder flower buds. Detected in the vacuoles and the cell wall of organic matter, calcium fulfills various functions in the secretory and structural processes of tissues. Consequently, in the present study, after the blooming phase (H4F), high calcium values in the cell walls of unripe green elderberries (H5FrV) are maintained, but with the cell wall softening in the fruit maturity phase (H7FrN), a decline occurs due to the different way of calcium binding in tissue.

Ripe fruit contain less calcium than green fruit; this is explained by the messenger function of calcium in cellular signal transduction, which involves accumulation in the initial developmental stages of the elderberry [46]. 

During the growth stages of the analyzed samples, magnesium registers higher values in the case of the wild variety. Edible *Sambucus nigra* L. flowers contain the highest concentration of Mg (27.966 mg/kg) and Se (1.481 mg/kg), while edible Haschberg flowers contain the highest concentration of K (438.245 mg/kg), Fe (8.52 mg/kg) and Cr (3.142 mg/kg).

The recommendations of Dietary Reference Intakes (DRIs), according to Regulation (EU)No1169/2011 [47] are as follows for an adult: magnesium (375 mg/day), selenium (55 μg/day), potassium (2000 mg/day), iron (14 mg/day) and chromium (40 μg/day); based on this, edible elder flowers (regardless of the form of growing) can represent acceptable organic sources for the daily mineral requirements. 

The variations in Mg content in the samples included in the study can be explained by the implication of magnesium in the middle of the porphyrin ring attributed to the primary stage of chlorophyll biosynthesis and in carbohydrate partitioning metabolism. The introduction of magnesium into the structure of porphyrin is the first step of chlorophyll biosynthesis. Major changes during the development of the elderberry have been observed for magnesium, which is the central atom of the chlorophyll molecule, along with iron and copper, ensuring chlorophyll synthesis functionality.

Having a large bioavailability in plants and being easy to transport to the constitutive parts of plant organs, sodium was identified 7.4 times more in green elderberries (N5FrV) than in green elder flower buds in the wild variety (N1MV). The variation of sodium in the analyzed samples ranges between 12.727–209.725 mg/kg. Compared to the study of some medicinal plants, sodium can have values from 37–5584 mg/kg [46].

Potassium in both varieties registers a considerable decrease after the blooming phase (N4F and H4F), followed by an ascending accumulation tendency.

The influence of the growth stages on the microelements identified (Fe, Cr, Cu, Mn, Se, Cd, Pb and Ni) indicates maximum values in the primary growth stages of the elderberry, like in the case of macronutrients. Only the metals Cd and Ni do not obey this rule, being predominant in the ripe fruit phase (N7FrN and H7FrN). The daily recommended amounts are small for micronutrients Fe, Cu and Mn (less than 100 mg/day), but the dietary plant sources rich in such micronutrients are important. This is explained by their involvement in the regulation of the nervous system function [46]. 

The growth stages of *Sambucus* have an impact on iron content, which is lower in the case of the cultivated genotype compared to the wild genotype, across all developmental stages (from buds to ripening).

The mineral elements Fe, Mn, Cu are actively involved in photosynthesis, in redox processes, Cu being a component of lignified tissue, also participating in pollen formation.

While copper, manganese and cadmium content have similar values in the different stages, nickel, lead, selenium, chromium and iron show oscillations during the ripening period (Table 3), in both varieties.

Chromium and manganese concentrations increased in the elderberry reddening period (N6FrR and H6FrR) and decreased in the maturity stage (N7FrN and H7FrN). Cadmium remained constant during elderberry development. The amount of accumulated heavy metals (Fe, Cr, Cu, Cd, Pb and Ni) is dependent on the development phase only in the case of the cultivated elderberry variety. Green Haschberg buds (H1MV) contain almost twice as much heavy metals as red fruit (H6FrR). 

However, Haschberg variety accumulated few heavy metals in the analyzed metals, a probable consequence of the culture area. It should also be noted that the Haschberg variety orchard is located in a rural area (Manau village), far from the heavy road traffic, which has a significant impact on the chemical composition of the elderberry. Studies report a significant correlation between the heavy metal content in the elderberry fruit and flowers in Poland and the influence of road traffic routes [25]. The proximity and intensity of the road traffic significantly contribute to the increase in the content of these elements in the analyzed raw material.

Even if *Sambucus Nigra Haschberg* fructus contains the highest amount of Cd (0.003 mg/kg H7FrN) and *Sambucus nigra* L. fructus has the highest Ni value (1.531 mg/kg N7FrN) of the elderberry growth stages, this does not prevent their optimal use in dietary or curative formulas. However, the values of heavy metals accumulated in the ripe fruit stage in both varieties do not exceed the limit of accepted concentrations (0.30 mg Cd/kg and 0.1–5 mg Ni/kg), recognized by the World Health Organization (WHO) [46]. 

Mainly analyzed for their commercial use, elderberry fruit have variable amounts of Ca (574–1528 mg/kg FW), K (2953–5494 mg/kg FW) and Na (13–146 mg/kg FW) [3]. 

The fruit at full maturity had optimal catalytic equipment for functioning even outside the plant, but the plant organs which appear throughout the genetic ripening process show an increase in growth hormone biosynthesis. Hence, a well-defined mineral variation results (12 minerals identified).

Among the identified minerals, cadmium was the heavy metal with the lowest values during the elderberry fructification period in both varieties. Lead was undetected in four growth stages in the cultivated variety (buds, green, red and ripe fruit).

In conclusion, the macronutrients and micronutrients predominating in different developmental stages of the studied varieties were: in green elder flower buds, calcium, copper and magnesium to an equal extent; in elder flowers, potassium and magnesium, iron, chromium and selenium. In green elderberries, the highest value of macronutrients was given by sodium, and among heavy metals, lead was well represented. In the case of red elderberries, the variation of microelements and macroelements did not reach a maximum value; in ripe elderberries, nickel was well represented. Therefore, the ripening phases highlight a new promising source of mineral elements in human nutrition.

Consequently, the individual synthesis of minerals is better represented in the cultivated variety than in the wild one. Thus, a good adaptation of the Haschberg variety to the conditions of the temperate zone and the implicit selection of the variety are observed.

### 4.6. Water and Ash

Humidity registered the lowest values in the pollen sample of the cultivated variety (16.880% H4Pln) and the highest values in wild elderberry inflorescences (83.61% N4F). In the case of elderberry fruit (N7FrN and H7FrN), humidity percentages can be comparable to those reported by Wu et al. [24]. 

Total ash content in the analyzed elderberry samples was extremely significant (*p* ≤ 0.001), dependent on the developmental phases (Table 4). Until the full blooming stage of the elderberry, ash had the highest values in pollen samples (2.93% HPln). Studies report that *S. nigra* L. inflorescences contain considerable amounts of total ash [14], being directly dependent on the amount of pollen in the constitution of the flower. The ash of green elderberry seeds (N5FrV, H5FrV), regardless of the site of growth, showed maximum values (about 5% of total ash content), indicating the fact that Transylvanian elderberry seeds can be considered valuable organic reservoirs of mineral elements. Not only the peduncle color) [1], but also total ash values change in the last developmental stage of the elderberry. In the peduncle/flower, mature/immature fruit relationship, the percentage of ash was higher in peduncles. From a biochemical point of view, elderberry flowers had a high-water content but flower buds had a higher total ash percentage.

## 5. Summary and Conclusions

The developmental stages of the elderberry have an extremely significant impact (*p* ≤ 0.001) on the various bioactive compounds identified and implicitly on antioxidant properties. TPC and antioxidant activity vary across the developmental stages of elderberries: in the early phase, green buds have high values that increase during the evolution of the bud, until before the flower onset. The elder flower blooming stage announces a decline in bioactive compounds in the elderberry; the decrease continues in the green and red elderberry stage, and there is a slight increase towards the full maturity of the fruit.

The pollen samples in the inflorescences of both *Sambucus* varieties were extremely rich in phenolic compounds, flavonoids, and had a high antioxidant action. Green elder flower buds contained significant amounts of chlorogenic acid, calcium, copper and magnesium. White elder flower buds represent the key developmental stage, due to the high values of total flavonoids, exceeding by far those of elderberry inflorescences. However, the elder flower blooming stage is important due to the total content of rutin, quercetin 3-glucoside, kaempferol-3-rutinoside; procyanidin dimer isomer 3; and among minerals, K and Mg. The variety influences total rutin content, because both the flowers and the buds of the Haschberg variety have a higher content of rutin than the wild variety. Therefore, new possible organic sources of rutin can be taken into consideration by various fields that promote a healthy life. During ripening of elderberries, especially in the last maturity stages, the peduncles acquire a distinct reddish color, which is specific to the elder plant, suggesting the abundant presence of polyphenols. During ripening, the TPC of the peduncle tends to increase from the red phase to the dark color phase, similarly to TPC values in the corresponding fruit. This is explained by the senescence of photosynthetic tissues in the peduncles. By comparing the green peduncles to the other analyzed samples, it can be observed that these might exceed even the elder flowers in terms of total flavonoid content. Peduncles represent the key biological material in this study, alongside elderberry seeds, through their mineral content as well. Green elderberries abound in catechin, epicatechin, procyanidin dimer isomer 2 and sodium. Red elderberries have moderate or low values of the studied compounds of the future ripe fruit. Ripe elderberries registered the highest concentrations of cyanidin 3-glucoside, procyanidin dimer isomer 1, cyanidin 3,5-diglucoside, cyanidin 3-sambubioside and cyanidin 3-sambubioside-5-glucoside.

The genotype proposed for medical applications is Haschberg genotype obtained in culture, a conclusion based on the high TPC values of pollen and fruit in the last developmental stages, the highest rutin concentrations in flowers, the calcium content in green buds, and K and Fe content in flowers.

This knowledge significantly contributes to creating the profile of bioactive compounds of the new natural resources of the common elderberry shrub from buds to ripening. The composition of vegetative stay of *Sambucus* recommends it as an important factor for nutrition and health.

## Figures and Tables

**Table 1:** Antioxidant activity, total flavonoids and phenols of different elderberry genotypes during developmental phases.

DPPH		Total Flavonoids		Total Phenols		Samples			Total Phenols		Total Flavonoids		DPPH	
(%)		(mg QE/100 g)		(mg GAE/100 g)					(mg GAE/100 g)		(mg QE/100 g)		(%)	
						**Buds**								
70.007 ± 0.000	e	1019.662 ± 0.001	d	565.445 ± 0.003	f	N1MV		H1MV	570.442 ± 0.00	f	1025.12 ± 0.00	d	71.005 ± 0.005	d
72.058 ± 0.033	d	1020.545 ± 0.007	c	648.063 ± 0.002	d	N2MV		H2MV	640.086 ± 0.00	e	1030.115 ± 0.007	c	70.057 ± 0.002	e
89.608 ± 0.001	b	1461.262 ± 0.038	b	883.066 ± 0.001	b	N3MG		H3MG	890.04 ± 0.00	b	1369.17 ± 0.084	b	90.004 ± 0.00	b
						**Flowers and Fruit**								
87.01 ± 0.002	c	973.178 ± 0.001	f	789.194 ± 0.001	c	N4F		H4F	770.144 ± 0.04	c	945.257 ± 0.001	e	89.047 ± 0.007	c
32.994 ± 0.007	h	488.895 ± 0.002	k	278.195 ± 0.003	m	N5FrV		H5FrV	269.877 ± 0.002	m	460.894 ± 0.002	l	30.588 ± 0.00	i
30.635 ± 0.002	i	304.548 ± 0.01	o	249.243 ± 0.003	n	N6FrR		H6FrR	290.586 ± 0.00	k	360.53 ± 0.001	n	45.222 ± 0.012	g
50.355 ± 0.004	f	502.012 ± 0.00	j	591.597 ± 0.00	e	N7FrN		H7FrN	679.254 ± 0.004	d	635.223 ± 0.00	h	65.965 ± 0.003	f
						**Pollen and Seeds**								
91.007 ± 0.000	a	2131.235 ± 0.005	a	1031.06 ± 0.014	a	N4Pln		H4Pln	1201.126 ± 0.002	a	2100.23 ± 0.001	a	92.007 ± 0.00	a
7.723 ± 0.002	s	273.693 ± 0.006	r	41.507 ± 0.001	s	N5SFrV		H5SFrV	39.124 ± 0.00	s	263.695 ± 0.003	r	6.398 ± 0.00	s
8.424 ± 0.004	r	253.83 ± 0.012	s	57.614 ± 0.001	r	N6SFrR		H6SFrR	56.241 ± 0.007	r	241.329 ± 0.004	s	7.984 ± 0.006	r
9.012 ± 0.012	p	292.803 ± 0.002	p	58.716 ± 0.002	p	N7SFrN		H7SFrN	59.367 ± 0.002	p	299.589 ± 0.00	p	9.125 ± 0.003	p
						**Peduncles**								
10.00 ± 0.007	o	425.557 ± 0.00	m	190.285 ± 0.005	o	N1PMV		H1PMV	192.214 ± 0.00	o	410.565 ± 0.002	m	11.254 ± 0.007	o
21.004 ± 0.001	l	578.778 ± 0.00	h	321.469 ± 0.001	j	N2PMV		H2PMV	310.787 ± 0.002	j	592.113 ± 0.002	i	19.588 ± 0.001	l
23.197 ± 0.001	k	562.074 ± 0.004	i	289.274 ± 0.002	k	N3PMG		H3PMG	279.214 ± 0.002	l	555.622 ± 0.002	j	22.564 ± 0.00	k
30.065 ± 0.007	j	739.255 ± 0.001	g	345.708 ± 0.002	h	N4PMF		H4PMF	360.705 ± 0.005	h	714.694 ± 0.005	g	29.633 ± 0.002	j
39.707 ± 0.002	g	986.828 ± 0.00	e	442.224 ± 0.00	g	N5PFrV		H5PFrV	429.963 ± 0.002	g	900.654 ± 0.00	f	42.565 ± 0.004	h
15.015 ± 0.004	m	455.485 ± 0.002	l	276.586 ± 0.007	m	N6PFrR		H6PFrR	249.347 ± 0.005	n	463.178 ± 0.00	k	16.174 ± 0.004	m
12.066 ± 0.004	n	317.819 ± 0.001	n	338.143 ± 0.002	i	N7PFrN		H7PFrN	323.415 ± 0.002	i	320.111 ± 0.00	o	13.257 ± 0.00	n
***		***		***			*(Sig.)*		***		***		***	

Values are expressed as mean. Values with different letters in the same column indicate statistically significant (Sig.) differences (Tukey’s test, *** extremely significant *p* ≤ 0.001; QE: quercetin equivalent; GAE: gallic acid equivalents; DPPH: 2,2-diphenyl-1-picrylhydrazyl).

**Table 2:** Representative flavonoids in methanolic extracts from *Sambucus nigra* L. and *Sambucus nigra Haschberg*, in different growth phases of the fruit.

Nr. Peak	1	2	3	4	5	6	7	8	9	10	11	12	13	Amount of (µg/mL)		
retention Time (min.)	3.01	3.47	4.02	10.27	11.55	12.09	12.37	13.38	13.74	15.67	16.40	16.81	17.67			
[M-H] + (m/z)	743; 581; 449; 287	581; 287	611; 449; 287	579	449; 287	355; 181	291	291	579	611; 303	465; 303	595; 287	579			
UV λ_max_ (nm)	520; 280	520; 280	520; 350; 280	279	520; 280	330; 280	280	280	279	360; 270	360; 270	356; 264	279			
**Samples**	**Cy 3-sam-5-gluc**	**Cy 3-sam**	**Cy 3,5-digluc**	**Procyan-DI**	**Cyan-G**	**Chlorog ac**	**Catech**	**Epicatech**	**Procyan-D II**	**Quercet-Ru**	**Q 3-gluc**	**K 3-rut**	**Procyan-D III**	**Anthocy**	**Flav**	**Hydroxyc ac**
														*1 + 2 + 3 + 5*	*4 + 7 + 8 + 9 + 10 + 11 + 12 + 13*	*6*
**N1MV**	40.783 ± 0.002 g	33.788 ± 0.002 g	28.891 ± 0.002 i	244.953 ± 0.002 g	Nd	5808.697 ± 0.002 c	125.758 ± 0.002 j	112.941 ± 0.002 j	116.145 ± 0.002 j	905.806 ± 0.002 h	73.45 ± 0.002 e	182.837 ± 0.002 d	317.368 ± 0.002 c	103.462 ± 0.006	2079.231 ± 0.016	5808.697 ± 0.002
**H1MV**	40.545 ± 0.002 h	Nd	29.53 ± 0.002 i	181.152 ± 0.002 j	Nd	10257.656 ± 0.002 a	129.718 ± 0.002 h	140.135 ± 0.002 g	142.739 ± 0.002 e	4057.00 ± 0.002 b	73.15 ± 0.002 f	410.596 ± 0.002 c	310.062 ± 0.002 d	70.075 ± 0.004	5444.552 ± 0.016	10,257.656 ± 0.002
**N4F**	33.508 ± 0.002 j	Nd	31.728 ± 0.002 g	259.168 ± 0.002 f	77.839 ± 0.002 h	4948.771 ± 0.002 d	160.652 ± 0.002 g	162.609 ± 0.002 e	139.775 ± 0.002 g	1983.462 ± 0.002 c	127.141 ± 0.002 b	520.236 ± 0.002 b	1827.588 ± 0.002 b	143.075 ± 0.006	5180.588 ± 0.016	4948.771 ± 0.002
**H4F**	37.916 ± 0.002 i	Nd	29.787 ± 0.002 h	358.277 ± 0.002 c	121.287 ± 0.002 g	7429.495 ± 0.002 b	195.378 ± 0.002 d	202.984 ± 0.002 b	147.205 ± 0.002 d	6178.463 ± 0.002 a	209.863 ± 0.002 a	1244.874 ± 0.002 a	2913.322 ± 0.002 a	188.99 ± 0.006	11,450.366 ± 0.016	7429.495 ± 0.002
**N5FrV**	53.048 ± 0.002 f	34.972 ± 0.002 f	31.843 ± 0.002 f	206.516 ± 0.002 i	228.418 ± 0.002 e	2072.697 ± 0.002 e	173.396 ± 0.002 f	191.229 ± 0.002 d	198.236 ± 0.002 c	1254.869 ± 0.002 f	51.402 ± 0.002 i	57.882 ± 0.002 g	95.691 ± 0.002 g	348.281 ± 0.008	2229.221 ± 0.016	2072.697 ± 0.002
**H5FrV**	55.598 ± 0.002 e	39.319 ± 0.002 e	35.78 ± 0.002 e	240.074 ± 0.002 h	177.161 ± 0.002 f	1186.13 ± 0.002 f	396.994 ± 0.002 a	205.707 ± 0.002 a	239.426 ± 0.002 a	1504.402 ± 0.002 d	37.304 ± 0.002 j	55.995 ± 0.002 h	106.498 ± 0.002 e	307.858 ± 0.008	2786.4 ± 0.016	1186.13 ± 0.002
**N6FrR**	92.338 ± 0.002 c	40.903 ± 0.002 d	76.132 ± 0.002 c	350.014 ± 0.002 d	277.996 ± 0.002 d	302.247 ± 0.002 i	191.222 ± 0.002 e	140.873 ± 0.002 f	142.164 ± 0.002 f	256.771 ± 0.002 i	73.984 ± 0.002 d	72.89 ± 0.002 e	98.916 ± 0.002 f	487.369 ± 0.008	1326.834 ± 0.016	302.247 ± 0.002
**H6FrR**	74.825 ± 0.002 d	41.469 ± 0.002 c	37.607 ± 0.002 d	259.423 ± 0.002 e	680.508 ± 0.002 c	884.757 ± 0.002 h	384.233 ± 0.002 c	200.235 ± 0.002 c	216.962 ± 0.002 b	1407.889 ± 0.002 e	65.738 ± 0.002 g	49.011 ± 0.002 j	Nd	834.409 ± 0.008	2583.491 ± 0.014	884.757 ± 0.002
**N7FrN**	260.971 ± 0.002 b	82.463 ± 0.002 b	156.729 ± 0.002 b	1361.398 ± 0.002 b	3045.063 ± 0.002 b	99.587 ± 0.002 j	129.298 ± 0.002 i	131.723 ± 0.002 h	127.479 ± 0.002 h	158.603 ± 0.002 j	61.958 ± 0.002 h	59.901 ± 0.002 f	Nd	3545.226 ± 0.008	2030.303 ± 0.014	99.587 ± 0.002
**H7FrN**	332.164 ± 0.002 a	121.412 ± 0.002 a	170.991 ± 0.002 a	1479.139 ± 0.002 a	4611.039 ± 0.002 a	923.757 ± 0.002 g	386.604 ± 0.002 b	118.635 ± 0.002 i	122.281 ± 0.002 i	948.865 ± 0.002 g	118.758 ± 0.002 c	51.443 ± 0.002 i	Nd	5235.606 ± 0.002	3225.725 ± 0.014	923.757 ± 0.002
(Sig.)	***	***	***	***	***	***	***	***	***	***	***	***	***			

Flavonoids in matrix type: cy 3-sam-5-gluc: cyanidin 3-sambubioside-5-glucoside; cy 3-sam: cyanidin 3-sambubioside; cy 3,5-digluc: cyanidin 3,5-diglucoside; procyan-DI: procyanidin dimer isomer1; cyan-G: cyanidin 3-glucoside; chlorog ac.: chlorogenic acid; catech: catechin; epicatech: epicatechin; procyan-D II: procyanidin dimer isomer 2; quercet-Ru: quercetin 3-rutinoside (rutin); q 3-gluc: quercetin 3-glucoside; k 3-rut: kaempferol-3-rutinoside;procyan-D III: procyanidin dimer isomer 3; anthocy: anthocyanins; flav: flavonols; hydroxyc ac: hydroxycinnamic acids. Values are expressed as mean of two replicates. Values with different letters in the same column indicate statistically significant differences (Sig.) (Tukey’s test, *** extremely significant *p* ≤ 0.001; 1—n.d., cannot be determined as there is no statistical difference between the results.

**Table 3:** Macroelements and microelements of Transylvanian elderberry during seven ripening phases. Results are given as mean ± SD.

Ca	K	Mg	Na	Samples	Fe	Cr	Cu	Mn	Se	Cd	Pb	Ni
298.336 ± 0.004 d	75.92 ± 0.042 f	24.165 ± 0.021 b	28.045 ± 0.007 i	**N1MV**	6.38 ± 0.028 e	0.685 ± 0.007 f	11.285 ± 0.021 c	30.835 ± 0.021 c	0.855 ± 0.021 f	0.001 ± 0.000 c	0.004 ± 0.000 b	0.415 ± 0.007 g
707.229 ± 0.001 a	15.945 ± 0.007 i	15.05 ± 0.014 e	97.638 ± 0.000 e	**H1MV**	1.185 ± 0.007 h	0.46 ± 0.014 i	16.115 ± 0.021 a	38.585 ± 0.007 a	0.915 ± 0.007 e	0.002 ± 0.000 b	Nd	0.397 ± 0.001 h
74.552 ± 0.000 i	175.447 ± 0.000 c	27.966 ± 0.000 a	114.398 ± 0.001 c	**N4F**	7.627 ± 0.0001 b	0.315 ± 0.007 j	9.925 ± 0.007 d	25.21 ± 0.000 g	1.481 ± 0.001 a	0.002 ± 0.000 b	0.001 ± 0.000 d	1.227 ± 0.000 d
608.674 ± 0.000 c	438.245 ± 0.000 a	7.089 ± 0.000 g	12.727 ± 0.000 j	**H4F**	8.52 ± 0.000 a	3.142 ± 0.003 a	6.891 ± 0.001 g	36.122 ± 0.002 b	0.562 ± 0.002 h	0.002 ± 0.000 b	0.005 ± 0.000 a	0.252 ± 0.000 i
255.789 ± 0.000 e	0.48 ± 0.000 j	10.744 ± 0.000 f	209.725 ± 0.000 a	**N5FrV**	7.183 ± 0.000 c	2.231 ± 0.001 c	7.711 ± 0.001 f	26.56 ± 0.000 f	1.191 ± 0.001 b	0.001 ± 0.000 c	0.005 ± 0.000 a	1.396 ± 0.000 c
678.972 ± 0.000 b	21.506 ± 0.000 h	6.707 ± 0.000 h	72.726 ± 0.000 g	**H5FrV**	1.224 ± 0.000 g	0.89 ± 0.000 d	6.71 ± 0.014 h	30.174 ± 0.005 d	0.221 ± 0.002 i	0.002 ± 0.000 b	Nd	1.475 ± 0.000 b
215.874 ± 0.000 g	53.087 ± 0.000 g	22.687 ± 0.000 c	141.004 ± 0.000 b	**N6FrR**	6.772 ± 0.000 d	2.78 ± 0.000 b	9.871 ± 0.001 e	27.394 ± 0.006 e	0.681 ± 0.001 g	0.002 ± 0.000 b	0.003 ± 0.000 c	0.421 ± 0.000 g
235.743 ± 0.000 f	257.261 ± 0.000 b	6.147 ± 0.000 i	109.292 ± 0.000 d	**H6FrR**	0.963 ± 0.000 i	0.63 ± 0.000 g	6.551 ± 0.001 i	20.561 ± 0.001 h	1.15 ± 0.000 c	0.002 ± 0.000 b	Nd	0.634 ± 0.000 f
156.476 ± 0.000 h	117.409 ± 0.000 e	21.714 ± 0.000 d	57.344 ± 0.000 h	**N7FrN**	5.034 ± 0.000 f	0.751 ± 0.001 e	13.212 ± 0.003 b	13.87 ± 0.000 j	0.705 ± 0.007 g	0.001 ± 0.000 c	0.001 ± 0.000 d	1.531 ± 0.000 a
71.239 ± 0.000 j	152.187 ± 0.000 d	2.618 ± 0.000 j	80.815 ± 0.000 f	**H7FrN**	0.951 ± 0.000 i	0.56 ± 0.000 h	5.37 ± 0.000 j	14.93 ± 0.000 i	1.09 ± 0.000 d	0.003 ± 0.000 a	Nd	0.909 ± 0.000 e
***	***	***	***	(S)	***	***	***	***	***	**	***	***

Calcium (Ca), Potassium (K), Magnesium (Mg), Sodium (Na), Iron (Fe), Chromium (Cr), Copper (Cu), Manganese (Mn), Selenium (Se), Cadmium (Cd), Lead (Pb), Nickel (Ni). Values with different letters in the same column indicate statistically significant differences (Sig.) (Tukey’s test, *** extremely significant *p* ≤ 0.001; 1—n.d., cannot be determined as there is no statistical difference between the results.

**Table 4:** Percentage of humidity and ash determined in wild and cultivated elderberry (*Sambucus nigra*) genotypes during growth phases.

Humidity (%)		Ash (%)		Samples			Humidity (%)		Ash (%)	
				**Buds**						
80.549 ± 0.014	de	2.034 ± 0.014	h	N1MV		H1MV	80.608 ± 0.014	d	1.269 ± 0.035	k
69.853 ± 0.707	k	1.768 ± 0.049	i	N2MV		H2MV	67.175 ± 0.0707	l	1.892 ± 0.035	hi
82.263 ± 0.707	b	1.605 ± 0.063	j	N3MG		H3MG	82.128 ± 0.777	bc	1.493 ± 0.014	j
				**Flowers and Fruit**						
83.619 ± 0.00	a	1.258 ± 0.063	k	N4F		H4F	82.28 ± 0.07	b	0.375 ± 0.026	o
80.133 ± 0.07	def	1.007 ± 0.007	l	N5FrV		H5FrV	76.86 ± 0.042	h	0.468 ± 0.035	o
78.592 ± 0.035	g	0.253 ± 0.042	p	N6FrR		H6FrR	75.614 ± 0.07	i	0.396 ± 0.014	p
77.304 ± 0.00	h	0.69 ± 0.014	n	N7FrN		H7FrN	66.65 ± 0.021	l	0.745 ± 0.049	mn
				**Pollen and Seeds**						
17.426 ± 0.021	s	2.402 ± 0.014	g	N4Pln		H4Pln	16.88 ± 0.00	s	2.935 ± 0.063	e
27.759 ± 0.021	r	5.572 ± 0.028	a	N5SFrV		H5SFrV	29.74 ± 0.028	p	4.57 ± 0.014	b
34.24 ± 0.028	o	3.703 ± 0.021	c	N6SFrR		H6SFrR	34.925 ± 0.02	1no	2.599 ± 0.014	f
35.985 ± 0.00	m	2.659 ± 0.096	f	N7SFrN		H7SFrN	35.14 ± 0.042	n	2.662 ± 0.042	f
				**Peduncles**						
79.746 ± 0.035	f	3.249 ± 0.028	d	N1PMV		H1PMV	79.855 ± 0.035	ef	3.278 ± 0.028	d
78.755 ± 0.021	g	2.981 ± 0.014	e	N2PMV		H2PMV	78.98 ± 0.014	g	2.69 ± 0.021	f
78.355 ± 0.049	g	2.572 ± 0.035	f	N3PMG		H3PMG	78.76 ± 0.007	g	3.187 ± 0.021	d
78.785 ± 0.021	g	1.848 ± 0.063	i	N4PF		H4PF	78.962 ± 0.028	g	1.566 ± 0.042	j
81.465 ± 0.035	c	2.687 ± 0.021	f	N5PFrV		H5PFrV	81.756 ± 0.035	bc	2.597 ± 0.014	f
75.06 ± 0.056	i	0.907 ± 0.014	l	N6PFrR		H6PFrR	75.5 ± 0.014	i	0.978 ± 0.028	l
74.98 ± 0.014	i	1.011 ± 0.007	l	N7PFrN		H7PFrN	73.665 ± 0.035	j	0.87 ± 0.028	lm
***		***			*(Sig.)*		***		***	

Values are expressed as mean. Values with different letters in the same column indicate statistically significant (Sig.) differences (Tukey’s test, *** extremely significant *p* ≤ 0.001).

## Data Availability

The data presented in this study are available on request from the corresponding author, due to privacy restriction.
